# In Situ versus Systemic Immune Response in the Pathogenesis of Cutaneous Leishmaniasis

**DOI:** 10.3390/pathogens13030199

**Published:** 2024-02-23

**Authors:** Augusto M. Carvalho, Rúbia S. Costa, Alexsandro Lago, Olívia Bacellar, Daniel P. Beiting, Phillip Scott, Lucas P. Carvalho, Edgar M. Carvalho

**Affiliations:** 1 Gonçalo Moniz Institute (IGM), Fiocruz, Salvador 40296-710, BA, Brazil; augustomarcelino1@hotmail.com (A.M.C.); rubia_suelymara@hotmail.com (R.S.C.); carvalholp76@gmail.com (L.P.C.); 2 Immunology Service, Professor Edgard Santos University Hospital Complex, Federal University of Bahia, Salvador 40110-160, BA, Brazil; alex-lago@hotmail.com (A.L.); olivinhaufba@gmail.com (O.B.); 3 Instituto Nacional de Ciência e Tecnologia em Doenças Tropicais (INCT-DT), Ministério da Ciência e Tecnologia e Inovação (MCTI), CNPq, Salvador 40110-160, BA, Brazil; 4 Department of Pathobiology, School of Veterinary Medicine, University of Pennsylvania, Philadelphia, PA 19104-4539, USA; beiting@vet.upenn.edu (D.P.B.); pscott@vet.upenn.edu (P.S.)

**Keywords:** cutaneous leishmaniasis, *Leishmania braziliensis*, immune response, immunopathology, IL-1β, granzyme B, IL-17

## Abstract

The role of the immune response in the pathogenesis of cutaneous leishmaniasis (CL) due to *Leishmania (Viannia) braziliensis* is predominantly carried out via blood cells. Here, we evaluate whether cytokine production by peripheral blood mononuclear cells (PBMCs) reflects what has been documented at the lesion site. The participants included 22 CL patients diagnosed with a positive PCR. PBMCs were stimulated for 72 h with a soluble leishmania antigen (SLA). Biopsies obtained from the edge of the ulcers were incubated for the same period. Cytokines in supernatants were assessed via ELISA. TNF, IL-1β, IL-6, IL-17, and granzyme B (GzmB) were higher in the supernatants of biopsies than in PBMCs, but IFN-γ was higher in the supernatants of PBMCs than in biopsies. There was a positive correlation between IFN-γ and TNF in PBMCs, and an inverse correlation between TNF and IL-10 in the cells from the lesion site. A strong correlation between IL-1β, IL-17, and GzmB was observed in the biopsies, and a positive correlation was detected between these cytokines and the lesion size. Our results indicate that the immune response in *L. braziliensis* lesions is different from that observed in peripheral blood, and our data suggest that in addition to IL-1β and GzmB, IL-17 participates in the pathology of CL.

## 1. Introduction

The interplay between infecting agents and host factors determines the control or progression of infectious diseases. The role of the immune response in the pathogenesis of leishmaniasis has been well studied, but there are still gaps in translating observed in vitro results to beneficial disease management [1]. In cutaneous leishmaniasis (CL), more than 10 different species of parasites of the genus *Leishmania* can cause human disease. Although cutaneous lesions caused by the different *Leishmania* species are similar, the pathogenesis may be quite different. While parasites are found in large numbers in the lesions caused by *L. guyanensis*, *L. mexicana*, *L. major*, and *L. tropica*, parasites are scarce in tegumentary leishmaniasis caused by *L. braziliensis* [2,3,4,5]. Moreover, while *L. tropica*, *L. major*, *L. peruviana*, *L. panamensis*, and *L. mexicana* cause only CL, *L. braziliensis*, *L. amazonensis*, and *L. guyanensis* may cause CL, mucosal leishmaniasis (ML), and disseminated leishmaniasis (DL). For example, in *L. guyanensis* infection, the parasite burden is associated with a down-modulated Th1 immune response and CD8^+^ regulatory T cells secreting IL-10 [6,7]. In contrast, in CL caused by *L. braziliensis*, patients have an immune response associated with protection. However, as *L. braziliensis* escapes from the host defense mechanisms, the parasites survive and an exaggerated immune response ends by causing pathology [8,9,10,11,12,13]. In patients with CL, immune response studies have predominantly been performed in peripheral blood mononuclear cells (PBMCs) or using histopathologic and immunochemistry analysis of biopsied tissues. These studies indicate that in contrast to experimental murine infections, susceptibility is not associated with a dominant Th2 response [8,14,15,16]. While IFN-γ is the principal cytokine that activates macrophages for leishmania killing, IL-10 allows for parasite proliferation and is associated with visceral leishmaniasis and diffuse cutaneous leishmaniasis [17,18,19,20]. In a few patients with CL due to *L. braziliensis*, the low production of IFN-γ is associated with large and atypical ulcers and therapeutic failure [21]. These studies have also shown the association between the systemic immune response and the severity of the clinical forms of *L. braziliensis* infection, as patients with ML and atypical CL have a higher lymphocyte proliferation and produce higher levels of pro-inflammatory molecules than cases with classical CL [8,13,20,22]. While pro-inflammatory cytokines are documented at high levels in all clinical forms of *L. braziliensis* infection, the regulatory cytokine (IL-10) rather than IL-4 or IL-5 plays a pivotal role in the modulation of the immune response. However, its production allows for parasite multiplication [8,23,24].

Studies on cytokine production and identification of cellular activation have also been performed with skin biopsies from leishmanial ulcers, and they have documented an association between cytokine expression and the activation of macrophages, CD8+ T cells, and NK cells with a pathology [11,25,26,27,28,29]. The aim of the present study was to perform a comparative analysis between the immune response observed in the supernatants of cells from the edge of *L.* (*V*.) *braziliensis* skin ulcers and that documented in the supernatants of PBMCs stimulated with SLA. There were important differences between the production of cytokines when these molecules were evaluated in the supernatants of biopsies rather than in the supernatants of the PBMCs, as well as strong correlations between an in situ production of some cytokines such as IL-1β, IL-17, and granzyme B (GzmB) with clinical features and CL pathology.

## 2. Study Design

This study was performed in order to compare the immune response observed from blood cells with that detected in the skin of the ulcers of CL patients. All of them (N = 22) presented classical ulcers of CL and had the diagnosis confirmed through the detection of the DNA of *L. braziliesis* by PCR or identification of amastigotes in histopathologic studies. They were of both genders, with an age > 18 < 60 years, who looked for medical attention at the Reference Center for Diagnosis and Treatment of Tegumentary Leishmaniasis from December of 2022 to June of 2023. This center, also known as the Health Post of Corte de Pedra, is located in Southeast of Bahia state in Brazil. The cytokines chosen for studying were associated with protection (IFN-γ, TNF) or the pathology (IL-1β, IL-17, GzmB) of CL due to *L. braziliensis*, and the size of the ulcers was considered a marker of pathology.

## 3. Materials and Methods

The tissue samples and peripheral blood used in the study were obtained from 22 participants of the study. Cytokines were assayed in the supernatants of PBMCs and skin biopsies. All immunologic evaluations were performed before therapy. This project was approved by the Ethical Committee of the Hospital Universitário Professor Edgard Santos, and all patients signed an informed consent form.

### 3.1. Soluble Leishmania Antigen (SLA) and Leishmania Skin Test (LST)

SLA was prepared as described elsewhere with a *L. braziliensis* isolate [30]. For the LST, 25 μg of SLA in 0.1 mL was inoculated intracutaneously in the forearm, and induration was measured 48 h post-inoculation. A positive LST was considered when the induration was equal to or greater than 5 mm.

### 3.2. Culture of PBMCs and Biopsied Skin Tissue

PBMCs were isolated from heparin-treated venous blood via Ficoll-Hypaque gradient centrifugation. After washing three times in 0.9% NaCl, the cells were re-suspended in an RPMI 1640 culture medium (GIBCO BRL, Grand Island, NY, USA) supplemented with 10% human AB serum, 100 IU/mL of penicillin and 100 μg/mL of streptomycin. Cells (3 × 10^6^/mL) were plated in 24-well plates and stimulated with SLA (5 μg/mL). After incubation for 72 h at 37 °C and 5% CO_2_, supernatants were collected and stored at −20 °C. Biopsies from *L. braziliensis* lesions performed with a 4 mm punch were adjusted by weight and cultured in RPMI media supplemented with 10% fetal bovine serum at 37 °C, and 5% CO_2_ for 72 h. As amastigotes are present in the tissue of CL patients and preliminary results did not show differences in cytokine production in unstimulated versus SLA-stimulated skin biopsies, all the biopsies were kept without a stimulus. The supernatants were collected and stored at −20 °C. Cytokine levels in culture supernatants from PBMC and CL lesions were determined via enzyme-linked immunosorbent assay (ELISA) (R&D Systems, Minneapolis, MN, USA), and the results were expressed as pg/mL. As there was no detectable production of cytokines in the unstimulated PBMC cultures, the data were not expressed in fold change.

### 3.3. Statistical Analysis

Comparisons between local and systemic immune response were performed by the Wilcoxon paired test, and comparisons between 2 groups of patients were performed by the Mann–Whitney test. Correlation between cytokines and clinical features was performed by a non-parametric Spearman correlation test. Correlation matrix and other analyses were conducted using GraphPad Prism version 10.0 for Windows, and differences were considered significant at *p* < 0.05.

## 4. Results

Table 1 shows the demographic and clinical features of the 22 patients. All had classical CL lesions with one or more well-limited ulcers with raised borders. The age ranged from 18 to 54 years, and 15 (68.2%) were male. Most of the cases looked for medical help less than sixty days from the onset of their symptoms, and 15 (68.2%) had only one lesion. The LST was positive in all patients.

### 4.1. Local and Systemic Production of Cytokines and Granzyme B in CL Patients

**Figure 1:**
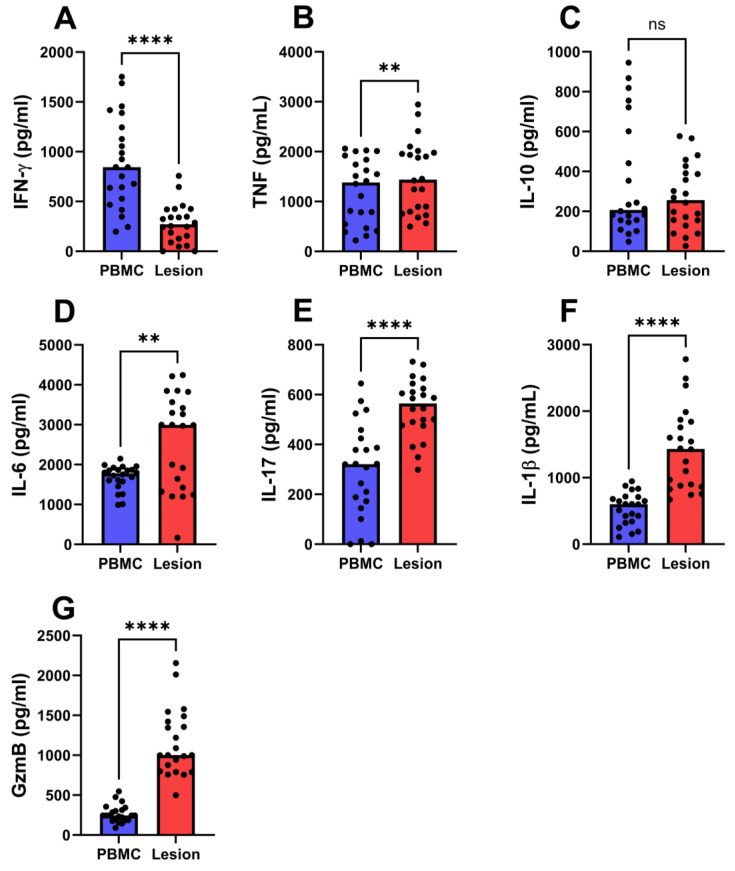
**Local and systemic cellular immune response in cutaneous leishmaniasis patients.** PBMCs and cells from the lesion of 22 CL patients were cultured in the presence of SLA (5 µg/mL) or without stimulation, respectively, for 72 h. Cytokine levels in culture supernatants were measured by ELISA: (**A**) IFN-γ, (**B**) TNF, (**C**) IL-10, (**D**) IL-6, (**E**) IL-17, (**F**) IL-1β, and (**G**) granzyme B. Circles represent individual values and bars represent median levels. ** *p* < 0.01, **** *p* < 0.0001.

CL caused by *L. braziliensis* infection is characterized by a strong inflammatory response that can control parasite replication but may also induce tissue damage [25,28]. Differences in the cellular immune response in peripheral blood and lesions are shown in Figure 1. Higher levels of IFN-γ were observed in PBMC cultures 844 pg/mL (198–1753) compared to CL lesions 271 pg/mL (0–758), while the systemic and local production of IL-10 were similar (Figure 1A,C). Alternatively, cell cultures from the lesions displayed higher levels of TNF, IL-6, IL-17, IL-1β, and GzmB than PBMC cultures (Figure 1D–G). Of note, the median values of IL-17, IL-1β, and GzmB in CL lesions were 1.7, 2.3, and 4.1 times higher than in peripheral blood, respectively.

**Figure 2:**
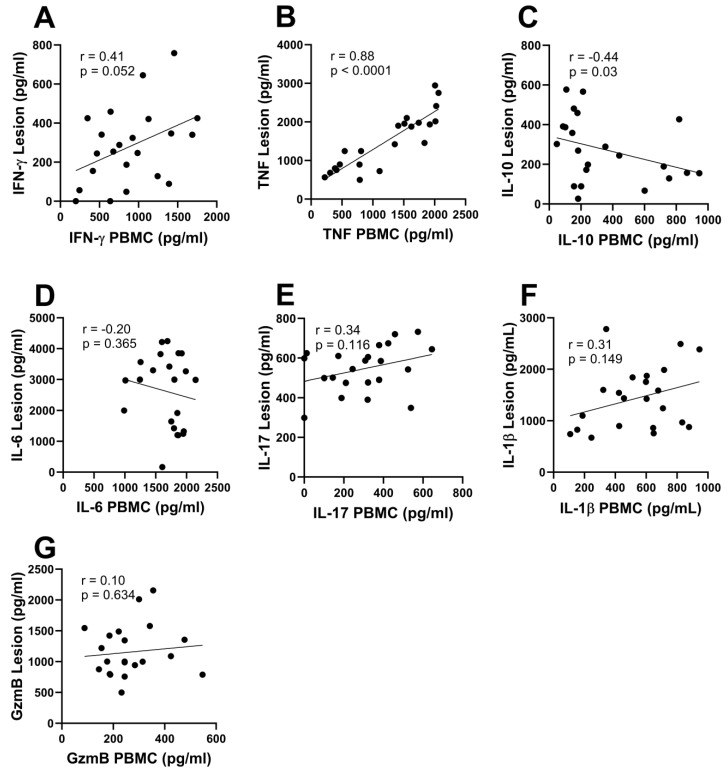
**Correlation between local and systemic immune responses**. PBMCs and cells from the lesion of 22 CL patients were cultured in the presence of SLA (5 µg/mL) or without stimulation, respectively, for 72 h, and cytokine levels in culture supernatants were measured by ELISA. Spearman correlation between in situ and systemic levels of (**A**) IFN-γ, (**B**) TNF, (**C**) IL-10, (**D**) IL-6, (**E**) IL-17, (**F**) IL-1β, and (**G**) granzyme B.

The correlations between the cytokine levels in the supernatants of biopsies with the ones observed in supernatants from stimulated PBMCs are shown in Figure 2. The only positive correlation was between TNF levels in peripheral blood and CL lesions (r = 0.88, *p* < 0.0001) (Figure 2B). Interestingly, we also observed a negative correlation between IL-10 levels in the two cell cultures (r = −0.44, *p* = 0.03) (Figure 2C).

### 4.2. Immune Response in L. braziliensis Lesions Correlates with Disease Severity of CL

**Figure 3:**
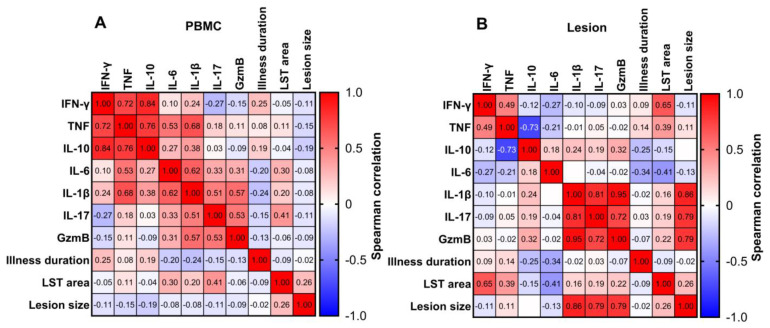
**Correlation matrix of the immune response and clinical features in cutaneous leishmaniasis patients.** Correlation matrix of systemic (**A**) and local (**B**) immune response and clinical characteristics. Squares represent individual cytokine values. Red squares depict a positive Spearman correlation, and blue squares depict a negative Spearman correlation. r values are shown inside the squares.

A correlation matrix showed the influence of the systemic and local immune response in CL pathogenesis (Figure 3). We observed the presence of two major clusters of cytokines positively correlated with each other. The first cluster contained IFN-γ, TNF, and IL-10 in PBMC cultures. Of note, IFN-γ and IL-10, as well TNF and IL-10 displayed strong positive correlations (r = 0.84, *p* < 0.0001; r = 0.76, *p* < 0.001), respectively. Furthermore, TNF was positively correlated with IL-6 and IL-1β. Alternatively, no correlation was detected between IFN-γ and IL-10 in CL lesions. The second cluster contained IL-1β, IL-17, and GzmB in *L. braziliensis* lesions. Importantly, we found a strong direct correlation among IL-1β and GzmB (r = 0.95, *p* < 0.0001) and IL-17 (r = 0.81, *p* < 0.0001). However, in PBMC supernatants, we only detected a weak positive association between the same molecules.

The Leishmania skin test (LST) is a delayed-type hypersensitivity (DTH) reaction to SLA. It has been associated with protection against *Leishmania* infection in subjects living in endemic areas who are exposed to infection but do not develop the disease [31,32]. Here, we found a positive correlation between LST and IFN-γ production in CL lesions (r = 0.65, *p* = 0.001), whereas no correlation was detected in peripheral blood.

**Figure 4:**
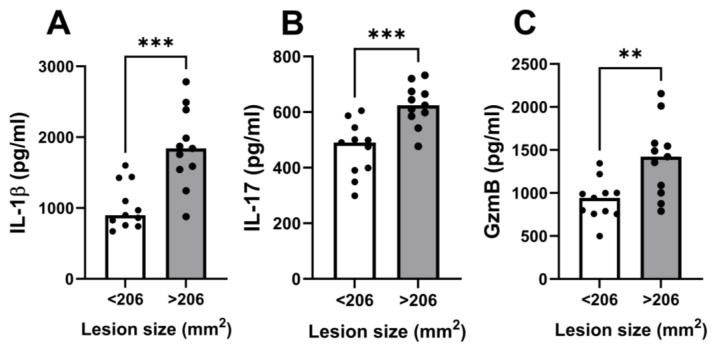
**In situ cytokine levels are associated with disease severity in CL patients.** Cells from the lesions of 22 CL patients were cultured without stimulation for 72 h. Cytokine levels in culture supernatants were measured by ELISA: (**A**) IL-1β, (**B**) IL-17, (**C**) granzyme B. Circles represent individual values and bars represent median levels. ** *p* < 0.01, *** *p* < 0.0005.

**Figure 5:**
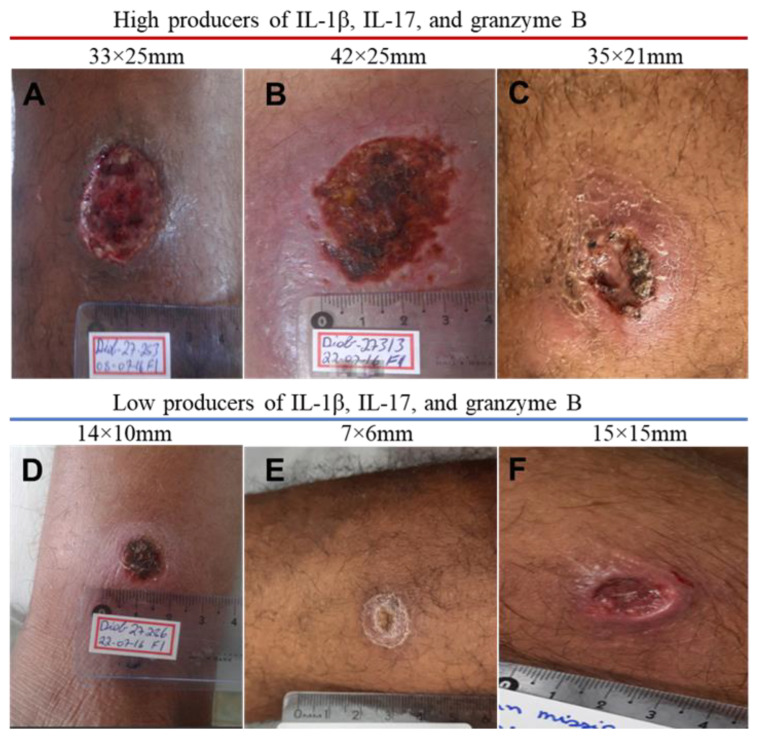
**Pictures of CL lesions from three high and three low producers of IL-1β, IL-17, and GzmB**. Figures A, B and C are from 3 patients who had high levels of IL-1β, IL-17 and granzyme B and had ulcers larger and more deep than the ones shown in Figures D, E and F that are from 3 patients with low production of these cytokines.

The ulcer clinically represents the pathology in CL, and larger lesions are linked to treatment failure [33]. We found a strong positive correlation between IL-1β, IL-17, and GzmB levels and lesion size. Notably, the strongest correlation between ulcer size and in situ cytokine production was detected with IL-1β (r = 0.86, *p* < 0.0001), followed by GzmB (r = 0.79, *p* < 0.0001) and IL-17 (r = 0.79, *p* < 0.0001). However, there was no correlation between lesion size and the same cytokines in PBMC cultures. We further stratified CL patients by median lesion size. Patients presenting an ulcer size larger than 206 mm^2^ displayed significantly higher levels of IL-1β, IL-17, and GzmB in lesion cultures compared to patients whose ulcers were smaller than 206 mm^2^ (Figure 4). Lastly, the representative pictures of lesions from high and low producers of IL-1β, IL-17, and GzmB are shown in Figure 5. In addition to larger ulcers, patients who were high producers of these cytokines presented deeper and more inflammatory lesions.

## 5. Discussion

Studies with whole blood cells, PBMCs, and even serum or plasma have made crucial contributions to diagnosing diseases and understanding the protection and the pathology associated with infectious diseases. In visceral leishmaniasis (VL) and diffuse cutaneous leishmaniasis, the impairment of the Th1 immune response in the pathogenesis of these diseases was clarified by studies in peripheral blood [18,19]. Asymptomatic or subclinical CL can be detected when whole blood cells or PBMCs are stimulated with SLA and produce IFN-γ [32]. Cytokine levels in the supernatants of PBMCs are also associated with clinical forms of the disease and treatment failure [8,21,22,34]. In CL, inflammatory responses develop to control parasite multiplication, but *L. braziliensis* can survive in a robust pro-inflammatory environment, and pro-inflammatory cytokines may provoke pathology [8,13,25,26]. More recently, it has been shown that pathology in CL is linked mainly to CD8 T cells, NK cells, NRLP3 inflammasome activation, and expression of IL-1β and GzmB [25,26,27,28,29].

However, does the immune response observed in the peripheral blood reflect what happens in the lesion? The present study addressed this question by determining cytokine levels in supernatants prepared from PBMC and skin biopsies of CL patients infected with *L. braziliensis*. There was no correlation between the immune response for most of the cytokines from the two sites. However, lesion size was strongly correlated with IL-1β, IL-17, and GzmB produced by cells from the edge of the ulcer. Such a correlation was absent or exhibited only a weak correlation between these same cytokines in peripheral blood. As lesion size is associated with treatment failure, these data indicate that IL-1β, IL-17, and GzmB production in lesion biopsies is a marker of disease severity and may be related to failure to respond to therapy.

The demographic and clinical features of the participants of this study were characterized by a predominance of young males, as has been observed in many studies. It is known that patients with a short illness duration, mainly diagnosed before the appearance of the ulcer, have a weaker immune response in PBMCs than patients with classical ulcers [13], but here, the duration of the illness less than 30 days in only two cases.

For most of the cytokines tested, their levels were higher in the supernatants of the biopsied skin than in PBMCs, which may be explained by the migration of antigen-reactive cells to the lesion site. However, the lower production of IFN-γ in the lesion site compared to that observed in the PBMCs was unexpected. It is known that CD4^+^, CD8^+^ T cells, NK cells, and double-negative T cells produce IFN-γ in CL [10,35,36]. The relatively low IFN-γ level in the skin may be due to a rapid uptake of IFN-γ by macrophages to increase their ability to kill *Leishmania*. Alternatively, a low amount of IFN-γ detected at the lesion site could be related to the small contribution of CD8^+^ and NK cells to produce this cytokine in the tissue, as these cells in CL lesions are predominantly cytotoxic rather than inflammatory [26,28,29]. Moreover, the decreased expression of IFN-γ may be due to the block of the NF-κB activation pathway limiting IFN-γ-stimulating factor (IRF-1) synthesis, as observed in cells infected with *L. amazonensis* or *L. mexicana* [37,38].

Contrary to IFN-γ, production of IL-1β, IL-6, IL-17, and GzmB levels were higher at the lesion site than in PBMCs. This agrees with the high expression of IL-1β, IL-6, and GzmB detected in the biopsies of CL lesions [25,28]. IL-17 is associated with chronic inflammatory and autoimmune diseases, but it can also act to benefit tissue health [39,40,41,42]. PBMCs of subjects with subclinical *L. donovani* infection produce IL-17 and IL-22, while these cytokines are not produced by cells from VL patients [42]. Regarding the role of IL-17 in the pathology of tegumentary leishmaniasis in CL, there is a strong correlation between the frequency of CD4+ IL-17+ T cells and the total number of cells in the inflammatory infiltrate in skin lesions [43]. Moreover, IL-17 induces GM-CSF synthesis and granulopoiesis, and neutrophilic infiltration is detected in necrotic tissue of ML lesions [44]. Here, a direct correlation between IL-17 production in skin biopsies and lesion size was documented.

Despite the observation that the levels of most cytokines were higher in the lesion, a correlation between the expression of the same cytokine in peripheral blood and tissue was expected. However, this was only observed with TNF and IL-10. Regarding the other cytokines tested, it is noteworthy that there was no correlation between the production levels of these molecules in PBMC and biopsy supernatants. This finding draws attention to the fact that CL’s immune response studies should preferably be carried out with biopsied tissue rather than with blood cells. In the present study, we confirmed a positive correlation between IFN-γ and TNF with IL-10 in peripheral blood [13,24]. However, there was no positive correlation between IFN-γ and TNF with IL-10 at the lesion site. The direct correlation between the production of pro-inflammatory and regulatory cytokines has been explained as an attempt of IL-10 to down-modulate the inflammatory response [8,13]. In such cases, the absence of a positive correlation between IL-10 not only with TNF and IFN-γ, but also with other pro-inflammatory cytokines at the lesion site suggests an impaired participation of IL-10 in the control of the inflammatory reaction resulting in tissue injury and ulcer development.

Significant correlations existed between the production of different cytokines and their levels of expression of peripheral blood and skin cells, and the LST and lesion size. A positive correlation between IFN-γ and TNF in the supernatants of PBMCs confirms the strong relationship between these two cytokines, as we have previously documented that the neutralization of one of these cytokines decreases the expression of the other [24]. The LST is an “in vivo” marker of a cellular immune response associated with protection [21,31], and a direct correlation between IFN-γ production and the size of the LST is expected. However, this correlation was only observed with IFN-γ production in lesion supernatants. As the DTH reaction is mediated by the migration of dermal or lymph node-reactive cells to the site of antigen injection, this may explain the lack of correlation between the size of the LST and IFN-γ production by blood cells.

The strong correlation between IL-1β and GzmB at the lesion site, and their correlations with lesion size agrees with the documentation of their role in CL pathology [25,27,28,45]. We also observed a strong correlation between the production of IL-1β and GzmB in the skin and the production of IL-17. It is known that IL-17 amplifies inflammation by enhancing IL-1β production [46,47,48]. Above, we commented on the role of IL-17 in the pathology of ML, but a relationship between IL-17 and CL pathology has not been noted. The observation here of a direct correlation between IL-17 with IL-1β and GzmB, and the strong positive correlation between IL-17 and lesion size supports the role of IL-17 in tissue damage in CL.

As both the production of cytokines at the lesion site and the size of the ulcers were quite variable, to better evaluate the association between IL-1β, IL-17, and GzmB with lesion size, we compared these cytokine levels in the supernatants of skin biopsies with the size of the ulcers. The median lesion size was 206 mm^2^, and the levels of IL-1β, IL-17, and GzmB were higher in patients who had larger ulcers. Moreover, in patients producing low levels of these cytokines the ulcers were superficial and less inflammatory, while in the high producers, the lesions were deeper with higher borders.

We realize that there are limitations in the comparisons of cytokines produced by cells in the supernatants of peripheral blood and biopsies. The number of cells in PBMC supernatants was 3 × 10^6^, while in biopsy supernatants, it ranged from 4 to 20 million. Additionally, in the lesions, there are many inflammatory and non-inflammatory cells that may influence the immune response. It is also known that the number of antigen-specific reactive cells in biopsies is higher than in PBMCs. However, our results clearly show important differences between the response of PBMCs and lesion tissue cells. Finally, the only positive association between cytokine production and the severity of the disease occurred with cells cultured from skin biopsies.

The present study indicates significant differences between cytokine production by peripheral blood cells and cells from the skin lesions of CL patients. Importantly, we showed that in addition to IL-1β and GzmB, IL-17 was associated with lesion size. Collectively, these results indicate that the expression of IL-1β, IL-17, and GzmB at the lesion site may be used as a marker of severity of CL and gives support to the use of chemotherapy associated with immunotherapies, with an emphasis on down-modulating the production of these cytokines in the treatment of CL.

## Figures and Tables

**Table 1:** Demographic and clinical features of cutaneous leishmaniasis participants of the study.

LTCP	Age (Years)	Gender	Illness Duration (Days)	# of Lesions	Lesion Size (mm)	LST * (mm)
27176	54	M	30	2	20 × 15	22 × 20
27182	24	F	35	1	25 × 20	11 × 10
27183	37	M	30	1	25 × 16	15 × 11
27186	52	M	30	4	10 × 10	17 × 17
27195	25	F	60	1	05 × 02	13 × 12
27253	24	M	30	1	33 × 25	17 × 17
27273	29	F	90	1	07 × 07	15 × 10
27274	36	F	30	1	50 × 45	20 × 18
27286	30	M	45	1	14 × 10	15 × 14
27298	33	F	30	1	05 × 05	20 × 15
27313	46	M	90	1	42 × 35	15 × 15
30040	20	F	30	2	20 × 18	11 × 11
30042	18	M	15	2	15 × 15	15 × 14
30964	17	M	30	1	27 × 20	15 × 15
30970	48	F	60	2	08 × 07	13 × 12
30988	28	M	90	3	35 × 21	15 × 15
30991	36	M	40	1	15 × 14	12 × 10
30995	53	M	30	1	31 × 17	17 × 14
30997	41	M	30	1	07 × 06	14 × 12
30998	19	M	17	1	14 × 10	10 × 08
40003	35	M	60	2	32 × 16	17 × 15
40010	18	M	30	1	15 × 15	17 × 15

* LST—Leishmania skin test.

## Data Availability

These data are available under the DOI: 10.6084/m9.figshare.25270054.
